# Treatment and survival of non-alcoholic steatohepatitis associated hepatocellular carcinoma

**DOI:** 10.1186/s12885-015-1197-x

**Published:** 2015-04-01

**Authors:** Arndt Weinmann, Yvonne Alt, Sandra Koch, Carina Nelles, Christoph Düber, Hauke Lang, Gerd Otto, Tim Zimmermann, Jens U Marquardt, Peter R Galle, Marcus A Wörns, Jörn M Schattenberg

**Affiliations:** 1 Department of Medicine I, University Medical Center Johannes Gutenberg University, Mainz, Germany; 2 Cirrhosis Center Mainz (CCM), University Medical Center Johannes Gutenberg University, Mainz, Germany; 3 Clinical Registry Unit (CRU), University Medical Center Johannes Gutenberg University, Mainz, Germany; 4 Department of Diagnostic and Interventional Radiology, University Medical Center Johannes Gutenberg University, Mainz, Germany; 5 Department of General, Visceral and Transplant Surgery, University Medical Center Johannes Gutenberg University, Mainz, Germany; 6 Department of Transplantation and Hepatobiliopancreatic Surgery, University Medical Center Johannes Gutenberg University, Mainz, Germany

**Keywords:** NASH, HCC, Overall survival, Clinical database

## Abstract

**Background:**

The incidence of non-alcoholic steatohepatitis (NASH) is increasing worldwide and a poorly defined subset of patients develops end-stage liver disease and hepatocellular carcinoma (HCC). Differences in the biological behaviour, tumour characteristics, associated risk factors, treatment outcomes and overall survival of patients with NASH-HCC remain poorly defined. The aim of this study was to determine and analyze these differences in a large clinical cohort to guide treatment decisions.

**Methods:**

1119 patients with HCC treated in an 11 year period at the University Medical Centre of the Johannes Gutenberg University Mainz were retrospectively analyzed.

**Results:**

Patients with NASH-HCC (n = 45) were older (67.6 vs. 65 years), had an increased frequency of the metabolic syndrome and complications with a higher incidence of obesity (31.1% vs. 14.7%), type II diabetes mellitus (66.7% vs. 37.85%), a higher rate of myocardial infarction (13.3% vs. 4.8%) and apoplectic stroke (8.9% vs. 2.1%) (all p < 0.05). Interestingly, liver function was preserved to a higher extent and MELD scores were significantly lower in NASH-HCC. Nonetheless, resection or orthotopic liver transplantation was performed only in 17.8% and 4.4% of NASH-HCC respectively. Overall survival was lower compared to HCC of other aetiologies. Independent of the underlying aetiology BMI exhibited a positive correlation with overall survival.

**Conclusion:**

Despite retained liver function, patients with NASH-associated HCC showed a decreased overall survival. With regards to the expected increasing prevalence of NASH, it will be necessary to improve screening and surveillance strategies to identify HCC in NASH early and improve survival.

## Background

Hepatocellular carcinoma (HCC) is the fifth most common cancer and the third leading cause of cancer-related deaths worldwide [1]. Globally the majority of HCC are associated with chronic viral hepatitis with a high prevalence in less industrialized countries mainly East Asia and sub-Saharan Africa. Over the last years, the incidence of HCC in these countries is decreasing while the incidence of HCC in developed countries has increased [1,2]. This trend is likely related to the increasing prevalence of the metabolic syndrome and the associated risk factors including insulin resistance and obesity [3]. These risk factors contribute to the development of non-alcoholic fatty liver disease (NAFLD) which has become the most prevalent liver disease world-wide [4]. The clinical spectrum of NAFLD ranges from isolated hepatic steatosis to non-alcoholic steatohepatitis (NASH), which is characterized by hepatic necroinflammation and varying degrees of fibrosis [5]. The estimated prevalence of NAFLD in the adult population ranges from 9-37% with strong cultural and geographic differences and the prevalence of NASH is estimated at 3-5% [6]. Although epidemiological studies have to determine the risk of disease progression, it has become obvious that chronic inflammation in NASH is a trigger that can lead to the development of HCC – even in the absence of cirrhosis in a yet poorly defined subset of patients [7]. Despite advances in screening and therapy for HCC the overall prognosis is poor with a 5-year-survival rate of 15% [8]. Treatment decisions for HCC are commonly based on the Barcelona Clinic Liver Cancer (BCLC) staging system which considers performance status, tumour size and location, extra hepatic spread and the underlying liver function [9]. For the multimodal treatment of HCC different surgical, interventional (radiological/sonographical) and non-interventional procedures have been established. Curative treatment options include resection, orthotopic liver transplantation (OLT) or locoregional therapies and are available for early tumour stages. For intermediate tumour stages (BCLC B), transarterial chemoembolization (TACE) with or without drug-eluting beads (DEB-TACE) and selective internal radiation therapy (SIRT) are used. In advanced tumour stages (BCLC C) systemic therapy with the multikinase inhibitor sorafenib represents the current standard in patients with compensated cirrhosis [10]. Best supportive care is recommended for end-stage HCC patients (BCLC D) [11].

The global increase of metabolic risk factors including diabetes and obesity will lead to an increasing prevalence of NASH and complications including cirrhosis and HCC [7]. Currently only few studies have explored disease characteristics, treatment and outcome of NASH-related HCC. The aim of this retrospective analysis was to evaluate differences in the epidemiology, risk factors, tumour characteristics, therapy and overall survival in patients with NASH-HCC in contrast to non-NASH aetiologies in a European cohort over a period of 11 years.

## Methods

### Patient characteristics

Patients with HCC who were referred to the University Medical Centre of the Johannes Gutenberg University Mainz between January 2000 and December 2010 were included in a clinical database after informed consent was given and analyses were performed retrospectively. The diagnosis of HCC was made according to the AASLD/EASL criteria [12]. All patients were classified according to the tumour node metastasis staging system of the UICC [13] and the stage of HCC was described according to BCLC-classification [12]. Survival data were acquired from clinical records or by contacting registration offices. Tumor-specific treatment and survival times were extracted from patient records. Diabetes mellitus, hypertension, hyperlipidemia and the metabolic syndrome were defined according to the definitions of the Joint Scientific Statement for Harmonizing the Metabolic Syndrome [14]. Laboratory results were obtained at the time of initial diagnosis of HCC and were considered missing if not available within a maximum of 90 days. Liver cirrhosis was determined based on histological features or clinical signs including ascites, hepatic encephalopathy, thrombocytopenia, splenomegaly, laboratory results indicating impaired liver function. NASH was defined according to the histological features of NASH, when biopsy results were available. Cryptogenic cirrhosis in the presence of metabolic risk factors and in the absence of significant alcohol consumption was considered as NASH as previously established [15].

### Ethical consideration

This study was carried out in compliance with the Helsinki Declaration (http://www.wma.net/en/30publications/10policies/b3/index.html). No formal ethics approval was required for this strictly retrospective study as was ruled by the local ethics committee (Ethik-Kommission der Landesärztekammer Rheinland-Pfalz).

### Data analyses and statistics

Data are given as median and range for numeric variables, or as counts and percentages for categorical variables. For statistical evaluation continuous variables are compared between groups of patients by the Mann–Whitney U test; categorical variables are compared using Fisher exact test or its equivalent for more than 2 categories. All calculations were done with R version 3.0.2. A p-value below 0.05 was considered significant.

## Results

### Clinical characteristic and complications of the metabolic syndrome in NASH-HCC

**Figure 1:**
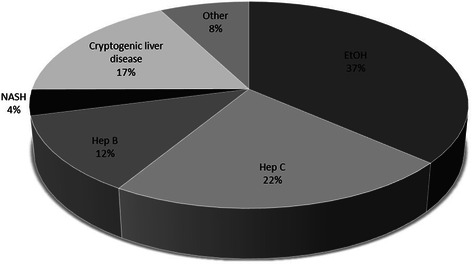
Aetiologies of HCC. Aetiologies of HCC in 1119 patients between 2000 and 2010.

A total of 1119 patients with HCC were included between 2000 and 2010 for further analysis. The median age for the entire cohort was 65.1 years (range 15.3-89.9), of which 82.6% (n = 924) were male and 98.3% (n = 1100) of Caucasian origin. Chronic viral hepatitis was the most frequent underlying cause of HCC (HBV 12.1%, HCV 22.7%), followed by alcohol-induced liver disease in 36%. In 17% of patients HCC developed in cryptogenic liver disease. Histological or morphologically confirmed NASH was identified in 4% (n = 45), which was validated by liver histology and after exclusion of significant alcohol consumption. Aetiologies of HCC are pictured in Figure 1. Baseline patient characteristics are summarized in Table 1. Patients with NASH-HCC were significant older (median age 67.6 versus 65 years, respectively; p = 0.007) and exhibited a lower predominance of male gender compared to non-NASH-HCC (77.8% (n = 35) vs. 82.7% (n = 888), p = 0.820).

**Table 1:** **Demographic data, prevalence of metabolic risk factors, complications and characteristics of liver function at time of initial HCC diagnosis**

Characteristics	NASH - HCC	Non-NASH - HCC	P
	(n = 45)	(n = 1074)	
Male gender*	35 (77.8)	888 (82.7)	0.820
Age at time of diagnosis^#^	67.6 (46.6-89.9)	65 (15.3-87.3)	**0.007**
Caucasian*	45 (100)	1055 (98.2)	1,000
Obesity*	13 (28.9)	158 (14.7)	**0.046**
BMI (kg/m^2^)^#^	29 (19.4-49.6)	26.6 (16.5-48.4)	**0.022**
Type II diabetes *	30 (66.7)	406 (37.8)	**0.024**
Hypertension*	32 (71.1)	485 (45.2)	0.060
Hyperlipidemia*	18 (40)	211 (19.6)	**0.016**
Myocardial infarction*	6 (13.3)	52 (4.8)	**0.035**
apoplectic stroke*	4 (8.9)	23 (2.1)	**0.025**
Cirrhosis*	35 (77.8)	858 (79.9)	1,000
Child-Turcotte-Pugh-Score*			
A	21 (46.7)	421 (39.2)	0.577
B	14 (31.1)	301 (28)	0.748
C	0	136 (12.7)	**0.012**
Bilirubin^#^	0.8 (0.3-4.8)	1.2 (0.2-78.3)	**<0.001**
INR^#^	1.1 (0.8-1.4)	1.1 (0.9-3.2)	**<0.001**
Creatinine^#^	1 (0.6-7.5)	0.9 (0.3-7.2)	0.420
Albumin^#^	35 (19–52.6)	34 (3–55.6)	0.173
Thrombocytopenia*	15 (33.3)	472 (43.9)	0.394
AFP^#^	96.9 (1.5-96611)	39 (0–624094.4)	0.722
MELD score^#^	9 (6–21)	10 (6–40)	**0.005**
Encephalopathy*	0	44 (4,1)	1.640
Ascites*	14 (31.1)	235 (21.9)	0.299
Varices*	10 (22.2)	342 (31.8)	0.420
Portal vein thrombosis*	13 (28.9)	222 (20.7)	0.290

*Data presented in (n [%]); ^#^Data presented in (median [range]); Bilirubin (normal range <1 mg/dL), Albumin (normal range 34–48 g/l, platelets count (normal range 150-450/nL), AFP (normal range <8 ng/mL). A p value p<0.05 was considered significant and is marked in bold.

The NASH-HCC group was defined by higher BMI and average BMI in NASH-HCC was (median [range]) 29 [19.4-49.6] kg/m^2^ vs. 26.6 [16.5-48.4] kg/m^2^; p = 0.022. Additionally, patients with NASH-HCC exhibited a higher prevalence of type 2 diabetes mellitus (66.7% vs. 37.8%, p = 0.024) and had a trend towards arterial hypertension (71.1% vs. 45.2%, p = 0.060). The rate of cardiovascular complications such as myocardial infarction (13.3% (n = 6) vs. 4.8% (n = 52), p = 0.035) and apoplectic stroke (8.9% (n = 4) vs. 2.1% (n = 23), p = 0.025) was significantly higher in patients with NASH-HCC **(**Table 1). Prevalence of metabolic risk factors and complications according to the underlying aetiology of HCC are displayed in Table 2.

**Table 2:** **Prevalence of metabolic risk factors and complications according to the underlying aetiology of HCC**

Characteristic	NASH	Alcohol	HBV	HCV
	(n = 45)	(n = 405)	(n = 135)	(n = 254)
Obesity	13 (28.9)	86 (21.2)	12 (8.9)	21 (8.3)
Typ II diabetes	30 (66.7)	200 (49.4)	39 (28.9)	66 (26)
Hypertension	32 (71.1)	200 (49.4)	57 (42.2)	96 (37.8)
Hyperlipidemia	18 (40)	92 (22.7)	28 (20.7)	45 (17.7)
Myocardial infarction	6 (13.3)	25 (6.2)	4 (3)	10 (3.9)
Apoplectic stroke	4 (8.9)	9 (2.2)	3 (2.2)	0 (0)
Thrombocytopenia	15 (33.3)	198 (48.9)	61 (45.2)	148 (58.3)

Data presented in (n [%]); platelets count (normal range 150-450/nL).

### Preserved hepatic function in NASH-HCC

Hepatic function in patients with NASH and non-NASH-HCC at the time of initial diagnosis are summarized and compared in Table 1. In contrast to the reports in the literature, there was a similar rate of cirrhosis in both groups (77.8% in NASH patients vs. 79.9%), with 21.2% of all HCCs developing in non-cirrhotic liver [16]. Patients with NASH-HCC exhibited a better hepatic function at the time of diagnosis. Correspondingly, the MELD score (median [range]: 9 [6–21] vs. 10 [6–40], p = 0.005), levels of bilirubin and INR were significantly lower in NASH-HCC. Regarding Child-Turcotte-Pugh Score (CTP) there was a lower rate of CTP stage C (p = 0.012) in NASH-HCC and a significant difference in platelet counts (NASH vs. non-NASH-HCC: 215/nl vs. 152/nl, p = 0.017). No differences with regards to ascites, portal vein thrombosis, oesophageal varices, presence of encephalopathy or levels of alpha-fetoprotein (AFP) at the time of diagnosis were observed.

### HCC characteristics in NASH and non-NASH origin

Histological confirmation of HCC was obtained in 87.1% of all patients. Tumour grading by the Edmondson-Steiner classification and BCLC tumour stages were comparable between NASH-HCC and HCC of other aetiology (Table 3). In NASH-HCC tumour size at the time of initial diagnosis exhibited a trend towards larger size compared to non-NASH-HCC (median [range]: 6 cm [1.5-16.5] vs. 4.8 cm [0–28], p = 0,176). Although no statistically relevant differences with regards to multifocal tumour spread was observed, this occurred in 80% of NASH-HCC and only in 69.7% of the non-NASH-HCC group. No difference was present with regard to lymph node metastasis while there was a trend towards more distant metastases in NASH-HCC.

**Table 3:** **Tumour characteristics and treatment in NASH vs non-NASH-HCC**

Characteristics	NASH - HCC	Non-NASH - HCC	P
	(n = 45)	(n = 1074)	
	(n [%])	(n [%])	
Histological confirmation of HCC	39 (86.7)	939 (87.4)	1.000
Grading			
G1	9 (20)	233 (21.7)	1.000
G2	20 (44.4)	438 (40.8)	0.781
G3	6 (13.3)	162 (15.1)	1.000
Median tumour size (cm)^#^	6 (1.5-16.5)	4.8 (0–28)	0.176
Metastases at initial diagnosis			
Lymph nodes	2 (4.4)	62 (5.8)	1.000
Distant	5 (11.1)	87 (8.1)	0.423
Morphology			
solitaire	9 (20)	319 (29.7)	0.324
multifocal	36 (80)	749 (69.7)	0.565
no data	0	6 (0.6)	1.000
BCLC at HCC diagnosis			
BCLC A	9 (20)	258 (24)	0.727
BCLC B	11 (24.4)	179 (16.7)	0.248
BCLC C	19 (42.2)	458 (42.6)	1.000
BCLC D	6 (13.3)	173 (16.1)	0.836
no data	0	6 (0.6)	1.000
UICC			
I	7 (15.6)	278 (25.9)	0.290
II	11 (24.4)	320 (29.8)	0.629
III	22 (48.9)	374 (34.8)	0.203
VI	0	0	1.000
Primary therapy			
Resection	8 (17.8)	213 (19.8)	1.000
OLT	0	43 (4)	0.407
OLT after bridging	2 (4.4)	188 (17.5)	0.054
TACE	19 (42.2)	503 (46.8)	0.785
RFA/PEI	2 (4.4)	55 (5.1)	1.000
Chemotherapy	1 (2.2)	22 (2)	0.615
Sorafenib	8 (17.8)	38 (3.5)	**<0.001**
Best supportive care	3 (6.7)	120 (11.2)	0.619
Overall survival (months)^#^	11.28 (0.7-127.6)	15.5 (0–131.3)	0.287

^#^Data presented in (median [range]). A p value p<0.05 was considered significant and is marked in bold.

### Differences in treatment and overall survival in NASH and non-NASH-HCC

**Figure 2:**
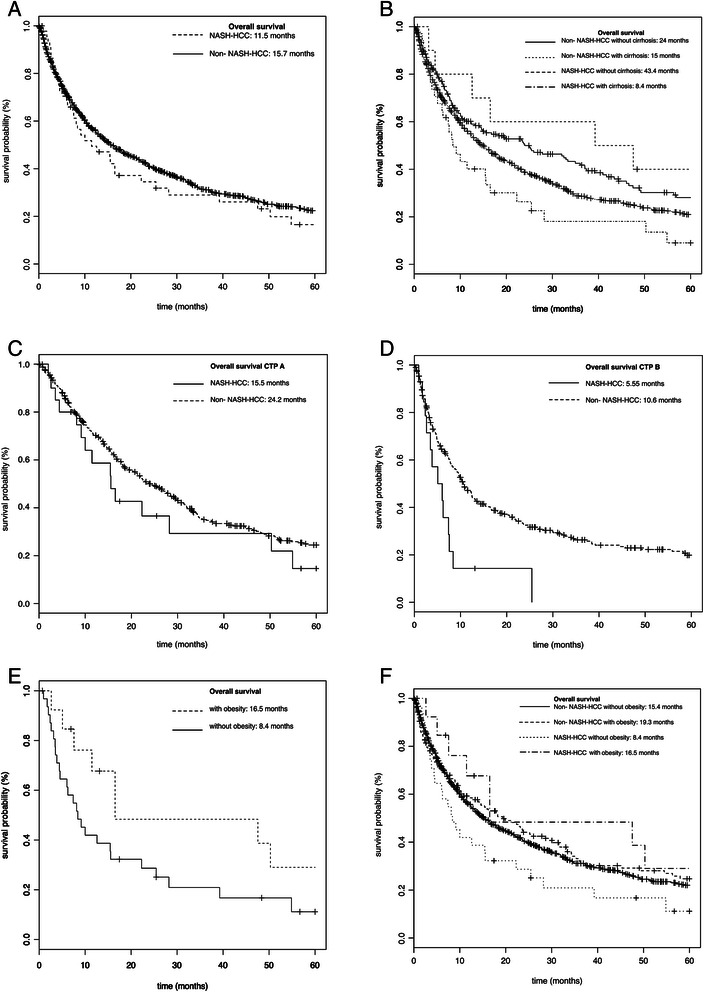
Kaplan-Meier survival curves. Kaplan-Meier survival curves comparing overall survival in NASH-HCC and non-NASH-HCC patients; **A** for all patients; **B** for all patients regarding presence of liver cirrhosis; **C** for all patients with Child Pugh stage A; **D** for all patients with Child Pugh stage B; **E** for all patients regarding obesity; **F** for NASH-HCC and non-NASH-HCC patients regarding obesity.

Primary therapy and overall survival (OS) are listed in Table 3. The most common treatment of HCC was transarterial chemoembolization (TACE) in both groups. No patient in the NASH-HCC group underwent orthotropic liver transplantation (OLT) as primary treatment while 4% of patients with non-NASH-HCC were transplanted. OLT following bridging therapy with TACE was performed in 4.4% of NASH and in 17.5% of non-NASH-HCC patients. Systemic therapy with sorafenib as a first line treatment was significantly more frequently performed in NASH-HCC (17.8% vs. 3.5%, p < 0.001). The median survival of all patients was 15.3 months (range 0–131 months). Notably overall survival (OS) in NASH-HCC was 4.22 months shorter compared to non-NASH-HCC (median [range]: 11.28 [0.7-127.6] vs. 15.5 [0–131.3], p = 0.287) (Figure 2). In HCC, OS is strongly depended on liver function. Imn the absence of cirrhosis, NASH-HCC patients showed a trend to an increased OS compared to non-NASH HCC patients (43.4 vs. 25 month, p = 0.748) (Figure 2). Both, compensated cirrhosis (CTP stage A) and decompensated cirrhosis (CTP stage B and C) were associated with a decreased survival in NASH-HCC compared to non-NASH-HCC. Patients with NASH-HCC in CTP stage A exhibited a decreased in OS (15.5 vs. 24.2 months, p = 0.268). The difference in OS in CTP stage B reached statistical significance (5.55 vs. 10.6 month, p < 0.05). In this clinical cohort there were no NASH-HCC patients with CTP stage C (Figure 2). A second factor that was identified to contribute to the OS in these patients was BMI. A higher BMI was associated with longer survival in all groups of HCC even independent of the underlying aetiology (Figure 2).

## Discussion

NAFLD – beeing the most prevalent liver disease in industrialized countries – can lead to NASH, where the emergence of HCC – even in the absence of cirrhosis – has been described [1]. Among all patients with NAFLD, the third leading cause of death is related to liver-specific causes [17] and HCC contributes significantly to this mortality [18]. Still, the underlying pathomechanism, the associated risk factors and incidence of NASH-HCC are poorly understood. In the current retrospective analysis, epidemiology, risk factors, tumour characteristics, therapy and overall survival in patients with NASH-HCC were evaluated in a large single-centre cohort consisting of 1119 HCC patients in Germany, which represents one of the most comprehensive cohorts in Germany [19]. In accordance to the literature, patients with NASH-HCC were significantly older compared to HCC of other aetiology [7]. However, the predominance of male gender commonly observed in HCC was less pronounced in the NASH-HCC group [20].

Since the definition of NASH-HCC included metabolic risk factors, the frequency of metabolic features and the frequency of obesity (BMI > 30 kg/m^2^) were significantly higher in this group. Recent studies have shown that a body mass index above 30 kg/m^2^ and the presence of type 2 diabetes are associated with an increased risk of HCC development [21,22]. In our analysis the prevalence of type 2 diabetes mellitus was almost two fold higher in NASH-HCC, comparable to the findings of Davila et al. [23]. Likewise, the prevalence of hypertension, as a common co-morbidity, was increased in NASH-HCC and a significant higher rate of myocardial infarction and apoplectic stroke was detected among NASH-HCC patients.

Recent studies showed an increasing number of patients with NASH-associated HCC in comparison to other underlying aetiologies and it has been suggested that NASH may become the most frequent cause of HCC in an era with improving therapeutic options for chronic viral hepatitis [1]. In the current analysis alcoholic liver disease (36%) was the most frequent cause of HCC, comparable to other investigations in Germany [24,25]. Interestingly, NASH-HCC accounted for only 4% of all HCC cases. The frequency of NASH-related HCC is likely influenced by the retrospective analysis which excluded all patients with any reported alcohol consumption. Thus this analysis likely excludes patients with NASH and a regular but low consumption of alcoholic beverages. Additionally, NASH as a cofactor in other underlying disease was not considered in this analysis. Several studies suggested that undiagnosed NASH is existent in patients with idiopathic or cryptogenic cirrhosis [2,7]. In the current cohort, cryptogenic cirrhosis accounted for 17% of cases, in analogy to findings in the literature, where a range between 6.9 up to 50% has been described in industrialized countries [7]. The prevalence of cryptogenic cirrhosis is clearly related to the quality of the data acquisition and handling and is also a potential confounder.

Superior hepatic function in NASH-HCC patients compared to other HCC is among the most relevant findings of the current analysis. Although there was no difference with regards to the prevalence of hepatic cirrhosis, more NASH-HCC patients presented in early CTP stage A or B and the MELD score was significantly lower. The difference between the two groups regarding the absolute MELD score with 9 vs. 10 seems low, but with regard to the range, the highest MELD score in NASH-HCC patients was 21 while in the other group it was 40. These findings are in accordance with data in the United States that found a lower MELD score in NASH-HCC patients [20].

The majority of HCC in this cohort was confirmed by histology. Although EASL and AASLD guidelines do not require a biopsy for diagnosis, these were frequently obtained following patient consent to (1) differentiate regenerative nodules from HCC and (2) to develop and identify prognostic marker. Thus we were able to assess tumour grading, where no significant difference between the two groups was observed. Interestingly, HCC was larger in the NASH-HCC group and we observed a trend towards multifocality and a higher rate of distant metastases at the time of diagnosis. In the literature, NASH-HCC has been typically described as large and well-differentiated at the time of presentation [7]. In HCC, curative treatment is only available in early stages, in which liver transplantation or resection is feasible. In the current analysis liver transplantation was not performed as primary therapy in any of the 45 cases of NASH-HCC. This might be related to the tendency of NASH-HCC to have a larger tumour size and a higher rate of multifocality at the time of diagnosis, possibly restricting surgical therapy. As discussed above, hepatic function was significantly better in these patients, and thus it is conceivable that the detection of cirrhosis and its complications were delayed. Other studies have made similar observations and suggested that NASH-related HCC may be diagnosed at a later time point and more advanced stage [26]. The differences in hepatic function also influenced the decision to initiate systemic treatment with sorafenib, which was significantly more frequently applied as primary treatment in our NASH-HCC patients. Sorafenib and transarterial chemoembolization are currently the only non-curative treatments that improve survival [27].

The results regarding the overall survival in NASH-HCC are inconsistent. In a recent study the overall survival following curative treatment approaches for HCC was increased in NASH compared to patients with HCV and/or alcoholic liver disease [20]. Wong et al. found that patients with NASH in the absence of HCC exhibited a better survival following OLT compared to patients with HCV or HCC of non-NASH origin [28], while Dyson and colleagues reported similar survival of NAFLD and other aetiologies for HCC [29]. In the current cohort, overall survival was shorter in NASH-HCC and a significant difference existed in the subgroup of patients with CTP B. It can be speculated that this decrease in survival is related to a delay in detection of the disease. Alternatively, these differences could be explained by different treatment strategies, since systemic therapy with sorafenib is currently only recommended for CTP A. Other explanations include the existence of co-morbidities and the higher age in NASH-associated HCC. A second central observation with regards to overall survival was the differences in patients with a higher BMI, which was protective. These observations are in contrast to several other studies, which reported a negative correlation of BMI and mortality in HCC [30,31]. Limitations of this analysis are related to the retrospective nature of the clinical cohort. Also, follow-up data regarding the cause of death was not completed available. In cases with complete death records mortality was related to complications of the underlying liver disease and tumour progression, rather than co-morbidities or cardiovascular disease.

## Conclusions

In conclusion, despite the growing prevalence of NASH, the frequency of NASH-HCC in a retrospectively derived European cohort between 2000 and 2010 is low. Nonetheless, metabolic risk factors are highly prevalent in these patients and a further increase is expected due to the strong association with obesity and diabetes [32]. Thus, screening efforts in NASH have to be intensified to avoid diagnosis at a late stage which excludes curative treatment options and exhibit a decrease in survival.

## Abbreviations

AFP: α-Fetoprotein
BCLC: Barcelona Clinic Liver Cancer
BMI: Body mass index
CTP: Child-Turcotte-Pugh Score
HCC: Hepatocellular carcinoma
INR: International Normalized Ratio
MELD: Model of end-stage liver disease
NAFLD: Non-alcoholic fatty liver disease
NASH: Non-alcoholic steatohepatitis
OLT: Orthotopic liver transplantation
OS: Overall survival
PEI: Percutaneous ethanol injection
RFA: Radiofrequency ablation
SIRT: Selective internal radiation therapy
TACE: Transarterial chemoembolization
UICC: Union Internationale Contre le Cancer
